# Hemoglobin α-derived peptides VD-hemopressin (α) and RVD-hemopressin (α) are involved in electroacupuncture inhibition of chronic pain

**DOI:** 10.3389/fphar.2024.1439448

**Published:** 2024-10-01

**Authors:** Xiaocui Yuan, Yixiao Guo, Huiyuan Yi, Xuemei Hou, Yulong Zhao, Yuying Wang, Hong Jia, Sani Sa’idu Baba, Man Li, Fuquan Huo

**Affiliations:** ^1^ Department of Physiology and Pathophysiology, School of Basic Medical Sciences, Institute of Neuroscience, Translational Medicine Institute, Xi’an Jiaotong University Health Science Center, Xi’an, China; ^2^ Key Laboratory of Environment and Genes Related to Diseases (Xi’an Jiaotong University), Ministry of Education, Xi’an, China; ^3^ Neuroscience and pathophysiology unit, Department of Human physiology, Faculty of Basic Medical Sciences, College of Health Sciences, Bayero University Kano, Kano, Nigeria; ^4^ Department of Neurobiology and Key Laboratory of Neurological Diseases of Ministry of Education, The Institute of Brain Research, School of Basic Medicine, Tongji Medical College, Huazhong University of Science and Technology, Wuhan, China

**Keywords:** Knee osteoarthritis (KOA), Electroacupuncture analgesia, VD-hemopressin (α), RVD-hemopressin (α), 26S proteasome, Chronic pain

## Abstract

**Introduction:**

Knee osteoarthritis (KOA) is a chronic degenerative bone metabolic disease that primarily affects older adults, leading to chronic pain and disability that affect patients’ daily activities. Electroacupuncture (EA) is a commonly used method for the treatment of chronic pain in clinical practice. Previous studies indicate that the endocannabinoid system is involved in EA analgesia, but whether endocannabinopeptide VD-hemopressin (α) and RVD-hemopressin (α) derived from hemoglobin chains are involved in EA analgesia is unclear.

**Methods:**

RNA-seq technology was used to screen which genes involved in EA analgesia. The expression of hemoglobin α chain and 26S proteasome were determined by Western blotting. The level of VD-hemopressin (α) and RVD-hemopressin (α) were measured by UPLC-MS/MS. Microinjection VD-Hemopressin (α), RVD-Hemopressin (α) and 26S proteasome inhibitor MG-132 into vlPAG, then observe mechanical and thermal pain thresholds.

**Results:**

Therefore, we used RNA-seq to obtain differentially expressed genes *Hba-a1* and *Hba-a2* involved in EA analgesia in the periaqueductal gray (PAG), which were translated into the hemoglobin α chain. EA significantly increased the expression of the hemoglobin α chain and the level of hemopressin (α) and RVD-hemopressin (α). Microinjection of VD-hemopressin (α) and RVD-hemopressin (α) into the ventrolateral periaqueductal gray (vlPAG) mimicked the analgesic effect of EA, while CB1 receptor antagonist AM251 reversed this effect. EA significantly increased the expression of 26S proteasome in KOA mice. Microinjection of 26S proteasome inhibitor MG132 before EA prevented both the anti-allodynic effect and upregulation of the concentration of RVD-hemopressin (α) by EA treatment and upregulated the expression of the hemoglobin α chain.

**Discussion:**

Our data suggest that EA upregulated the concentration of VD-hemopressin (α) and RVD-hemopressin (α) through enhancement of the hemoglobin α chain degradation by 26S proteasome in the PAG, then activated the CB1 receptor, thereby exerting inhibition of chronic pain in a mouse model of KOA. These results provide new insights into the EA analgesic mechanisms and reveal possible targets for EA treatment of chronic pain.

## 1 Introduction

Knee osteoarthritis (KOA) is a chronic condition characterized by knee joint degeneration that usually occurs from middle to older ages. In clinical practice, patients with KOA may experience joint pain, stiffness, and functional limitation associated with inflammation and effusion in the knee, which may affect their quality of life (Luan et al., 2021). Previous studies proved that electroacupuncture (EA) is effective in relieving chronic pain in KOA patients and animal models of KOA (Lv et al., 2019; Yuan T. et al., 2018). Accumulating evidence indicates that the classical lipophilic endocannabinoid arachidonic ethanolamine anandamide (AEA) and 2-arachidonoyl glycerol (2-AG) are involved in EA analgesia (Chen et al., 2009; Jiang et al., 2022; Yuan et al., 2018b). In addition, recent studies have shown that apart from the classical lipophilic endocannabinoid AEA and 2-AG, a class of cannabinopeptide ligands derived from hemoglobin α and β chains were isolated and identified from rodent brains and were found to have different affinity and selectivity for CB1 and CB2 receptors. Moreover, they have also been found to play certain regulatory roles in pain, feeding, learning, and memory (Leone et al., 2018; Zhang et al., 2016; Zheng et al., 2018). However, it is unclear whether cannabinopeptide ligands derived from hemoglobin chains are involved in EA analgesia.

Gomes et al. identified VD-hemopressin (α) and RVD-hemopressin (α) peptides derived from the hemoglobin α chain in the mouse brain using a peptide omics strategy, and *in vitro* functional experiments have shown that VD-hemopressin (α) and RVD-hemopressin (α) act as selective agonists for CB1 receptor, sustain the increase the release of intracellular Ca^2+^, and lead to increase ERK phosphorylation levels, suggesting that VD-hemopressin (α) and RVD-hemopressin (α) are cannabinopeptide ligands (Gomes et al., 2009). Previous studies have shown that both lateral ventricle and intrathecal injection of VD-hemopressin (α) can induce CB1 receptor-mediated analgesia in a test of acute pain caused by photothermal tail flick (Han et al., 2014; Zheng et al., 2017). In one study, VD-hemopressin (α) was injected into the lateral ventricle in pathological models of postoperative pain, formalin-induced inflammatory pain phase Ⅰ, and visceral pain, and a dose-dependent analgesic effect was observed (Zheng et al., 2017). These results indicate that VD-hemopressin (α) has an analgesic effect in the lateral ventricle and the intrathecal space. The ventrolateral periaqueductal gray (vlPAG) is a key structure in the modulation of pain, and our previous studies have also found that the vlPAG is involved in EA analgesia (Yuan et al., 2018c). However, whether VD-hemopressin (α) and RVD-hemopressin (α) are involved in EA analgesia in the vlPAG needs to be further explored.

VD-hemopressin (α) and RVD-hemopressin (α) are considered non-classical neuropeptides. Unlike classical neuropeptides, they are produced in the brain as neuropeptides derived from cytosolic proteins (Gelman and Fricker, 2010), but how they are produced is still unclear. In erythrocytes, hemoglobin is degraded by the proteasome after its ubiquitination or oxidation (Pacifici, Kono, and Davies, 1993; Wenzel and Baumeister, 1993). The proteasome is a large proteolytic complex ubiquitously distributed among mammalian cells, including neuronal cells (Tai and Schuman, 2008). Furthermore, the 26S proteasome is known to generate peptides ranging in length from 3 to 22 amino acids (Kisselev et al., 1999), consistent with the size of VD-hemopressin (α) and RVD-hemopressin (α). Therefore, one possible mechanism is that VD-hemopressin (α) and RVD-hemopressin (α) may be produced through 26S proteasome degradation of the hemoglobin α chain in neurons of the mouse brain.

In the present study, we determined whether the hemoglobin α chain is involved in the EA-mediated inhibition of chronic pain in a mouse model of KOA. Then, we examined whether VD-hemopressin (α) and RVD-hemopressin (α) are involved in the mechanism of EA-mediated inhibition of chronic pain. We also explored whether EA inhibited chronic pain by promoting 26S proteasome degradation of the hemoglobin α chain to produce VD-hemopressin (α) and RVD-hemopressin (α).

## 2 Methods and materials

### 2.1 Animals

Experiments were performed on 8-week-old male C57BL/6 J mice weighing 22–25 g (Medical Experimental Animal Center of Xi’an Jiaotong University). Male mice are preferred in our studies to eliminate variability introduced by hormonal cycles in female mice. Mice were housed in groups of 6 per home cage (32 cm × 21.5 cm × 17 cm) and had free access to food and water. All mice were maintained under controlled temperature (25 ± 2°C), humidity (50% ± 10%), and with a 12:12 h light/dark cycle (lights on at 8:00 a.m.). The study was conducted in compliance with the ethical guidelines of the International Association for the Study of Pain (Zimmermann, 1983) and approved by the Biomedical Ethics Committee of the Xi’an Jiaotong University Health Science Center (Animal committee number: 2019–841). Based on previous experience and pre-experiments, we estimated the sample size based on at least 80% power and α = 0.05. Each group used in the RNA-seq and quantitative polymerase chain reaction experiments contained three mice. In the Western blotting experiments, the number of mice in each group was 3–4. In experiments that analyzed the level of VD-hemopressin (α) and RVD-hemopressin (α), the number of mice in each group was 4–6. In the nociceptive behavioral tests, the number of mice in each group was 5–11.

### 2.2 Induction of knee osteoarthritis (KOA)

The mouse model of KOA was established following previously described procedures (La Porta et al., 2013). After mice were briefly anesthetized with isoflurane, 5 µL of monosodium iodoacetate (5 mg/mL; MIA; Sigma, UK) was injected into the left knee joint cavity. The concentration of MIA selected in this study can cause histological (Yuan T. et al., 2018) and pain behavioral changes (Harvey and Dickenson, 2009) in mice. MIA was dissolved in sterile saline. The control group animals were given 5 µL of sterile saline.

### 2.3 EA treatment

In the EA group, EA stimulation was performed on the left hindlimb of the mice, “Neixiyan” (Ex-LE4) and “Dubi” (ST35), from the second day of establishing the KOA model. The EA frequency was 2 Hz, the intensity was 1 mA, and the wave width was 0.1 m. EA was performed once every other day for 4 weeks, and each treatment lasted for 30 min. The present study opted for the parameter selection of EA according to our previous research (Yuan et al., 2018b).

The needles were inserted into the Ex-LE4 and ST35 points, respectively, 2–3 mm deep, corresponding to how humans would be treated. Ex-LE4 and ST35 are below the knee joint, located at the depression on the left and right sides of the patella. Ex-LE4 and ST35 were chosen because their use frequency is the highest in KOA, and they are specific acupoints for treating knee problems (Ng, Leung, and Poon, 2003; Selfe and Taylor, 2008).

### 2.4 RNA-seq analysis

Differential expression genes in the PAG of mice were determined by RNA-seq. By virtue of no difference in nociceptive threshold between the KOA group and the sham EA group (needling without electricity) in previous studies (Yuan et al., 2022; Yuan et al., 2018c), the mice that were used for the differential gene expression analysis were assigned to the CON, KOA, and EA groups. Nine mice were deeply anesthetized with 3%–5% isoflurane; the mice were then decapitated, and the PAG was dissected. The brain was removed from the skull and placed on a plate with the ventral surface up. The midbrain was taken and evenly cut into three subsections. The PAG was obtained with a 20G needle solution from the middle subsection of the midbrain and then placed into All Protect™ Nucleic Acid and Protein Stabilization Reagent for Animal Tissue (Beyotime Biotechnology, Shanghai, China).

Total RNA was isolated from the PAG specimens using a TRIzol reagent (Invitrogen, United States) according to the manufacturer’s protocol. Half of the RNA was used for sequencing analysis, and half was used for RT-qPCR validation. The RNA purity, concentration, and integrity were assessed by an RNA6000 Nano Reagents Port 1 Assay Kit of the Agilent Bioanalyzer 2100 system (Agilent Technologies). The final cDNA was sent for library preparation, and sequencing was performed by Wuhan BGI Technology Co., LTD., Wuhan, China (www.bgitechsolutions.com). We estimated the expression levels for all transcripts using the RSEM tool and calculated the expression levels for mRNAs in terms of fragments per kilobase of transcript per million mapped reads (FPKM). The differentially expressed genes (DEGs) were selected by the Noiseq method (comparison between every two groups) with fold change ≥2 and diverge probability ≥0.8. Clustering analysis of DEGs was performed with cluster (de Hoon et al., 2004; Eisen et al., 1998) and Java Treeview (Saldanha, 2004) software according to the CON-VS-KOA.KOA-VS-EA cluster plans for DEGs.

### 2.5 Quantitative polymerase chain reaction

The RNA-seq data were confirmed through RT-qPCR. cDNA was synthesized using the ReverTra Ace-a-TM (Toyobo, Japan) according to the manufacturer’s protocol. RT-qPCR analyses for mRNA were performed in a CFX96 system (Bio-Rad, UK) using the SYBR Green PCR amplification reagent (Toyobo, Japan), and the relative expression levels were quantified using CFX Manager software. β-Actin was used as the reference gene for normalization. The relative differences were expressed as the fold-matched control values calculated using the comparative cycle method (2^−△△CT^). A list of primers of selected genes is shown in Supplementary Table S1.

### 2.6 Nociceptive behavioral tests

Mechanical allodynia and heat hyperalgesia were also demonstrated in the hind paw of animals with KOA (Fernihough et al., 2004; La Porta et al., 2013). Mice were habituated to the testing environment for 30 min at least 3 days before testing. The baseline nociceptive thresholds were tested for 3 days before the MIA injection, and the average thresholds of 3 days were calculated as the baseline. After KOA induction, the nociceptive thresholds were tested once every other day, starting from the 18th day to 4 weeks.

The up-down method was implemented for the mechanical allodynia test (Chaplan et al., 1994). Mice were placed into a customized cage individually, and mechanical allodynia was determined with a series of von Frey filaments (Stoelting, Kiel, WI, United States). The area tested was the central plantar surface of the left hind paw, and von Frey filaments were applied perpendicularly to the plantar surface and maintained for 6 s until an S-shape formed. Sharp withdrawal, immediate flinching, or the raising, removal, shaking, or licking of the hind paw was considered a positive response. The test was repeated twice with a 5-min interval, and the average value was calculated.

The thermal test was performed by observing the paw withdrawal latency (PWL) to noxious heat with a model 336 analgesia meter (IITC, Inc., Life Science Instruments, Woodland Hills, CA, United States). A cut-off time of 20 s was used to avoid tissue damage to the animals’ paws. The test was repeated five times, and the mean value was calculated.

### 2.7 Analysis of VD-hemopressin (α) and RVD-hemopressin (α) by ultra-high performance liquid chromatography–tandem mass spectrometry (UPLC-MS/MS)

The PAG tissues were removed 4 weeks after vehicle or MIA injection. The PAG tissues were weighed, and eight times that weight of acetonitrile (ACN) was added and homogenized, after which ultrasonic treatment was performed for 20 min. After centrifugation at 13,000 rpm for 10 min, the supernatant was removed and filtered with a 0.22 μm filter membrane, followed by quantification with LC-MS/MS. Analyses were conducted on UPLC (I-Class)-MS (XEXO TQ-MS) and MassLynx V4.1 workstation. The parameters of mass spectrum detection of components to be measured were source voltages 3.00 KV; source temperature: 450°C; gas flow: 800 L/Hr; cone: 50 L/Hr. Analytical LC separations were performed on a Waters ACQUITY UPLC BEH-C18 (2.1*50 mm, 1.7 μm) with a flow rate of 0.3 mL/min and column temperature of 35°C using a gradient of ACN (eluent B) and water (eluent A), both containing 0.1% formic acid. The gradient was as follows: 2% eluent B for 1.0 min; 2%–100% B from 1.0 to 2.0 min and held at 100% from 2.0 to 3.5 min. From 3.5 to 4.0 min, the column was re-equilibrated to 2% B and conditioned from 4.0 to 6.0 min at 2% B (Bauer et al., 2012).

### 2.8 Western blotting

The PAG was removed as described above and put immediately into Allprotect™ Nucleic Acid and Protein Stabilization Reagent for Animal Tissue (Beyotime Biotechnology, Shanghai, China). The PAG tissues were then processed for protein extraction and Western blotting using the procedure described in detail in our previous study (Yuan et al., 2022; Yuan et al., 2021). After measuring protein concentrations using the BCA Protein Assay Kit (Beyotime Biotechnology, Shanghai, China), the samples were separated with 10% or 12% denaturing SDS-PAGE and transferred to a polyvinylidene fluoride (PVDF) membrane. PVDF membranes were blocked in TBST (pH 7.6, containing 0.1% Tween 20% and 5% non-fat milk) for 1 h at room temperature. Subsequently, the PVDF membranes were incubated with rabbit monoclonal anti-hemoglobin α antibody (1:1000, Abcam, UK), rabbit polyclonal anti-proteasome 26S S2 antibody (1:2000, Abcam, UK), or mouse monoclonal anti-β-actin antibody (1:5000; Proteintech, USA) at 4°C overnight. After washing with TBST, the blots were then incubated with the horseradish peroxidase-conjugated anti-rabbit or anti-mouse secondary antibodies (1:5000; EMD Millipore, Darmstadt, Germany). Protein bands were detected using an ECL kit (ECL-plus, EMD Millipore, Darmstadt, Germany) and captured using the Champchemi system with the SageCapture software (Sagecreation Service for Life Science, Beijing, China). The band intensity was quantified and analyzed using ImageJ software. The protein levels were quantitated relative to β-actin. Finally, the relative protein expression was normalized to the respective control group.

### 2.9 Intracerebral guide cannula placement

Mice were anesthetized with 2% sodium pentobarbital (50 mg/kg, intraperitoneal) and implanted with a guide cannula (RWD, China) 0.5 mm above the right vlPAG (AP: −4.8 mm, LM: +0.5 mm from midline, DV: −2.8 mm, from the skull surface) according to a mouse atlas. Once the animals recovered from anesthesia, sodium penicillin was administered (0.2 million units/day for 3 days, intraperitoneally) to prevent wounds and intracerebral infections. The animals were carefully nursed and fed in clean cages for at least 7 days to recover from the surgery.

### 2.10 Drug administration

On the 18th day after KOA induction, an injection cannula, connected to a 1 µL microsyringe, was extended 0.5 mm beyond the tip of the guide cannula for drug microinjection into the vlPAG. The drugs were dissolved in saline or 10% DMSO and then slowly infused through the microsyringe at a constant speed over a 1 min period. The injection cannula was left at the injection site for an additional 1 min to allow for complete diffusion of the injected drug. Drugs used in this study include the m-RVD-hemopressin (α) and m-VD-hemopressin (α), synthesized by Nanjing Synpeptide Biological Co., Ltd., which were dissolved in the 0.9% saline. MG132 (Z-Leu-Leu-Leu-al), a 26S proteasome inhibitor (Merck Millipore, United States) and AM251, a CB1 receptor selective antagonist (MCE, United States) were dissolved in the 10% dimethyl sulfoxide (10% DMSO; Sigma-Aldrich).

Drug doses were chosen according to previous studies (Han et al., 2014; Zheng et al., 2018) and our preliminary experiments. We used VD-hemopressin (α) at a concentration of 10 nmol in our study because it had been reported that intracerebroventricular administration of VD-hemopressin (α) produced a dose-dependent antinociception, and 10 nmol VD-hemopressin (α) also has an analgesic effect (Han et al., 2014; Zheng et al., 2018). RVD-hemopressin (α) was administered in various doses (1 nmol, 2 nmol, 5 nmol). MG132 (0.4 μg) was administered for 30 min before EA treatment (Yang et al., 2008). AM251 (20 nmol) was administered for 10 min prior to VD-hemopressin (α) and RVD-hemopressin (α) injections (Han et al., 2014). Equal volumes of 0.9% saline or 10% DMSO were injected into the vlPAG as vehicle controls.

### 2.11 Statistical analysis

All data were expressed as the means ± standard error (SEM). Behavioral data were analyzed using two-way repeated-measures ANOVA, followed by a Bonferroni *post hoc* analysis of multiple comparisons. Other data were analyzed by one-way ANOVA followed by a Newman–Keuls *post hoc* test with a two-tailed hypothesis or t-tests with a two-tailed hypothesis. All analyses were performed using the GraphPad Prism 9.0 software. The criterion for statistical significance was set as *p* < 0.05.

## 3 Results

### 3.1 RNA-seq screened differentially expressed genes *Hba-a1* and *Hba-a2* involved in EA analgesia

**FIGURE 1:**
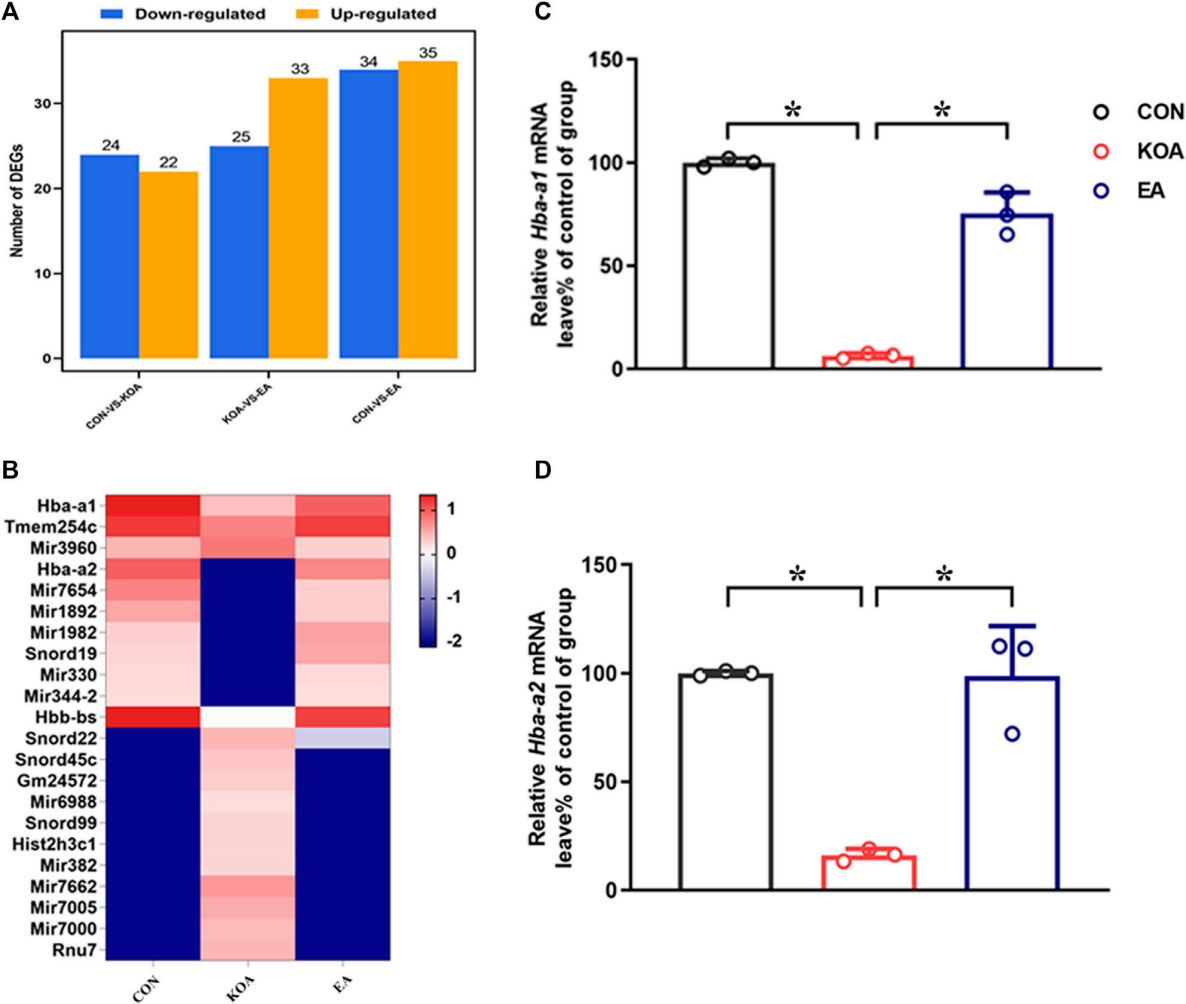
Screening differentially expressed genes (DEGs) *Hba-a1* and *Hba-a2* involved in EA analgesia in the periaqueductal gray. **(A)** DEG statistics. The *X*-axis represents pairwise comparisons, and the *Y*-axis shows the number of screened DEGs. The blue bars denote downregulated genes, and the orange bars denote the upregulated ones. **(B)** Heatmap of DEGs in the KOA, EA, and control groups. **(C)** Summary data show the effect of KOA and EA on the level of the *Hba-a1* in PAG tissues. **(D)** Summary data show the effect of KOA and EA on the level of the *Hba-a2* in PAG tissues. Data are expressed as means ± SEM (n = 3 mice in each group). *p < 0.05.

We used RNA-sequencing (RNA-seq) to compare gene alternations in the EA, KOA, and control groups. As shown in Figure 1A, compared with the control group, 24 genes were downregulated, and 22 genes were upregulated in the KOA group, and 34 genes were downregulated, and 35 genes were upregulated in the EA group. Moreover, compared with the KOA group, 25 genes were downregulated, and 33 genes were upregulated in the EA group. Genes with similar expression patterns usually have the same functional correlation. Cluster analysis showed that 22 genes were significantly altered, including 10 genes that were downregulated in the KOA group while upregulated in the EA group and 12 genes that were upregulated in the KOA group while downregulated in the EA group (Figure 1B).

Among these DEGs, the gene *Hba-a2*, which encoded the hemoglobin α chain, has the largest fold change. Interestingly, recent studies have shown that a class of cannabinopeptide ligands derived from hemoglobin α play certain regulatory roles in pain, feeding, learning, and memory (Leone et al., 2018; Zhang et al., 2016; Zheng et al., 2018). In addition, the gene *Hba-a1,* which also encoded the hemoglobin α chain, was found to have changed by more than threefold compared with the control group. Therefore, we chose *Hba-a1* and *Hba-a2* to further explore in this study. RT-qPCR was used to verify that observation, and it was found that the expressions of *Hba-a1* and *Hba-a2* in the PAG of KOA mice were significantly downregulated compared with the control group, and EA significantly increased the expression of both *Hba-a1* and *Hba-a2*, which was consistent with the results of RNA-seq (*p* < 0.05; Figures 1C, D).

### 3.2 EA reversed the reduction of hemoglobin α-chain expression in the PAG of KOA mice

**FIGURE 2:**
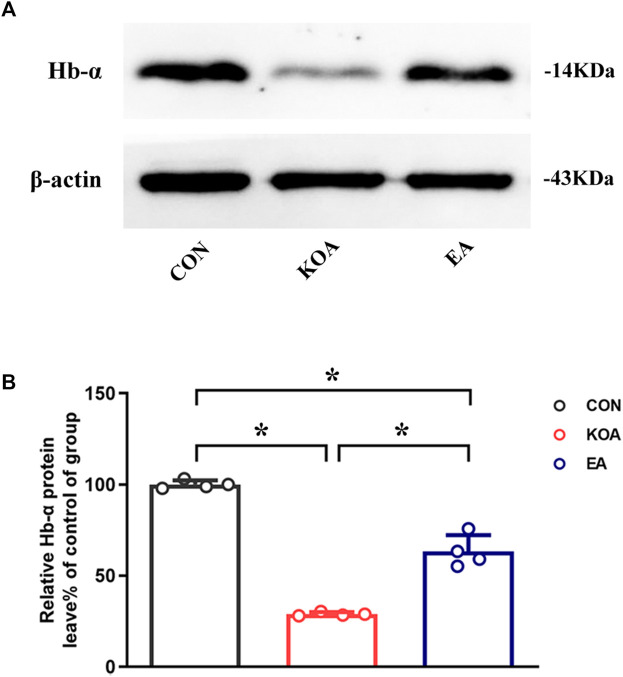
Quantitative analysis of the protein level of hemoglobin α-chain in the PAG tissues. **(A)** The representative gel image shows the protein level of the hemoglobin α-chain in PAG tissues obtained from the control (CON), KOA, and KOA treated with EA groups. β-actin was used as a loading control. The protein band at 14 kDa corresponds to the hemoglobin α-chain. **(B)** Summary data show the effect of KOA and EA on the protein level of the hemoglobin α-chain in PAG tissues. Data are expressed as means ± SEM (n = 4 mice in each group). *p < 0.05.

Because *Hba-a1* and *Hba-a2* encode the hemoglobin α chain, we used the Western blotting technique to observe the protein level of the hemoglobin α chain in the PAG. The hemoglobin α chain protein bands were present in the PAG tissues (Figure 2A). The hemoglobin α chain protein level in the PAG was significantly lower than that in the control group 4 weeks after KOA induction (*p* < 0.05; Figure 2B). EA treatment at 2Hz + 1 mA significantly increased the hemoglobin α chain level in the PAG compared with that in the KOA group (*p* < 0.05; Figure 2B), indicating that the hemoglobin α chain is involved in EA analgesia.

### 3.3 EA reversed the reduction of VD-hemopressin (α) and RVD-hemopressin (α) concentration in the PAG of KOA mice

Several studies have identified that VD-hemopressin (α) and RVD-hemopressin (α) peptides derived from the hemoglobin α chain in mouse brain and produced analgesic effects mediated by CB1 receptor (Gomes et al., 2009; Han et al., 2014; Zheng et al., 2017). Therefore, we determined whether EA increases the concentration of VD-hemopressin (α) and RVD-hemopressin (α) in the PAG.

**FIGURE 3:**
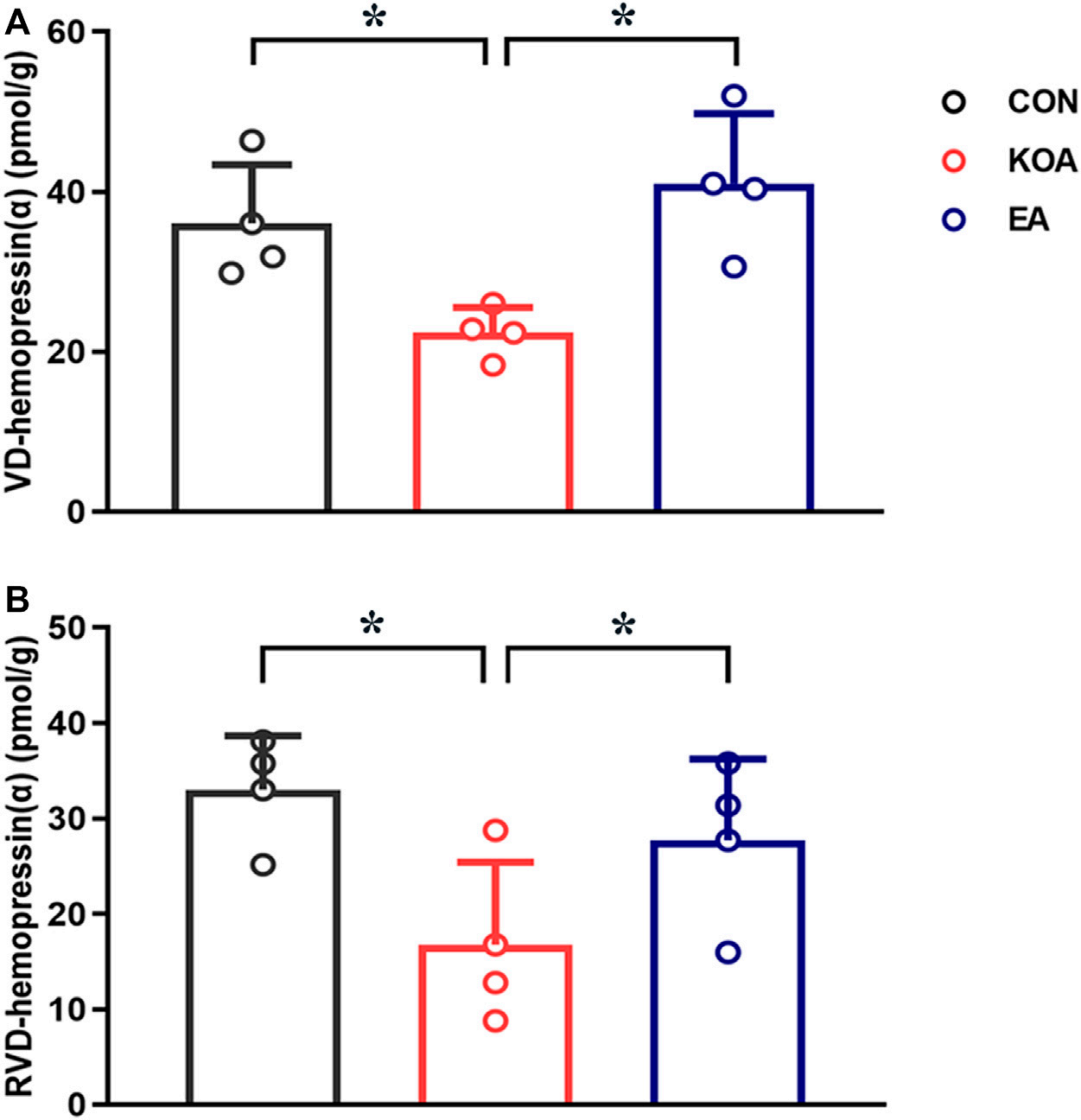
Quantitative analysis of the VD-hemopressin (α) and RVD-hemopressin (α) concentrations in the PAG. **(A)** Summary data show the effect of KOA and EA on the concentration of VD-hemopressin (α) in the PAG. **(B)** Summary data show the effect of KOA and EA on the concentration of RVD-hemopressin (α) in the PAG. Data are expressed as means ± SEM (n = 4 mice in each group). *p < 0.05.

The VD-hemopressin (α) and RVD-hemopressin (α) were detected in the PAG in all three groups 4 weeks after KOA induction. Compared with the control group, the VD-hemopressin (α) and RVD-hemopressin (α) levels in the PAG were significantly reduced in KOA mice (*p* < 0.05; Figures 3A, B). EA significantly increased the VD-hemopressin (α) and RVD-hemopressin (α) levels in the PAG of KOA mice (*p* < 0.05; Figures 3A, B).

### 3.4 Microinjection of VD-hemopressin (α) into the vlPAG mimicked the EA effect on pain hypersensitivity

**FIGURE 4:**
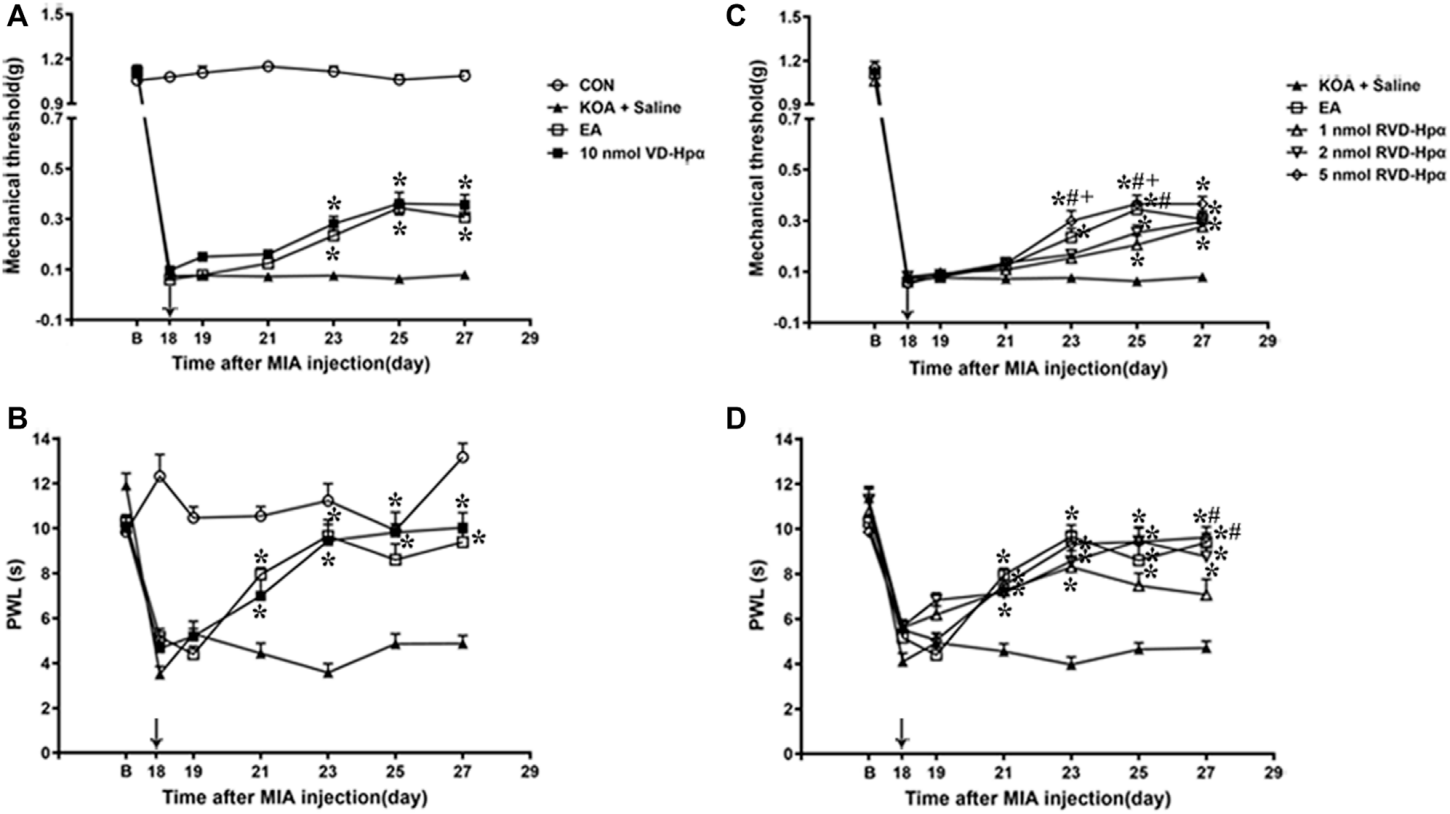
Time course of the effect of VD-hemopressin (α) and RVD-hemopressin (α) on pain hypersensitivity in KOA mice. **(A, B)** Time course of tactile threshold in response to von Frey filaments or a noxious heat stimulus in CON, KOA + saline, EA, and 10 nmol VD-hemopressin (α) mice. **(C, D)** Time course of the effect of different concentrations of RVD-hemopressin (α) on tactile and thermal withdrawal thresholds of KOA mice. VD-hemopressin (α) or RVD-hemopressin (α) was microinjected into the vlPAG, or the mice received EA stimulation starting from 18 days after MIA injection, once every other day for five times, as indicated by the black arrow. Data are expressed as means ± SEM (n = 8–11 mice in each group). *p < 0.05, compared with the KOA + Saline group; #*p* < 0.05, compared with the 1 nmol RVD-hemopressin (α) group; + *p* < 0.05, compared with the 2 nmol RVD-hemopressin (α) group.

Chronic pain was found to be established on the 17th day after KOA surgery, according to our previous research (Yuan. et al., 2018). In line with this, we microinjected VD-hemopressin (α) and RVD-hemopressin (α) into the right vlPAG on the 18th day after KOA induction, once every other day for five times. There was no difference in the baseline mechanical withdrawal threshold and thermal withdrawal latency between different groups before MIA injection. Microinjection of saline into the vlPAG did not influence the mechanical withdrawal threshold and thermal withdrawal latency induced by MIA (*p* > 0.05; Figure 4).

Microinjection of VD-hemopressin (α) (10 nmol/0.5 µL) into the vlPAG, contralateral to the affected knee, markedly increased the mechanical withdrawal thresholds of KOA mice (*p* < 0.05), as well as the EA group (*p* < 0.05). As shown in Figure 4A, the time course curves (i.e., CON, KOA + Saline, EA, and 10 nmol VD-hemopressin (α) groups) were significantly different between treatments (F_(3, 280)_ = 1489.06; *p* < 0.0001), across times (F_(6, 280)_ = 392.57; *p* < 0.0001), and for their interactions (F_(18, 280)_ = 54.7; *p* < 0.0001). Further analyses showed that the mechanical thresholds in the VD-hemopressin (α) group were significantly higher than those in the KOA + Saline group from the 23rd to the 27th day after KOA induction (*p* < 0.05), as well as those in the EA group (*p* < 0.05). No significant difference was observed between the VD-hemopressin (α) group and the EA group from the 23rd to the 27th day after KOA induction (*p* > 0.05; Figure 4A).

Microinjection of 10 nmol VD-hemopressin (α) into the vlPAG also significantly increased the thermal withdrawal latency of KOA mice, as well as the EA group (*p* < 0.05). As shown in Figure 4B, the time course curves (i.e., CON, KOA + Saline, EA, and 10 nmol VD-hemopressin (α) groups) were significantly different between treatments (F_(3, 196)_ = 1259.61; *p* < 0.0001), across times (F_(6, 196)_ = 358.24; *p* < 0.0001), and for their interactions (F_(18, 196)_ = 49.49; *p* < 0.0001). Further analyses showed that the mechanical thresholds in the 10 nmol VD-hemopressin (α) group were significantly higher than those in the KOA + Saline group from the 21st to the 27th day after KOA induction, as well as those in the EA group (*p* < 0.05). No significant difference was measured between the 10 nmol VD-hemopressin (α) group and EA group from the 21st to the 27th day after KOA induction (*p* > 0.05; Figure 4B), indicating clearly the mimicking capacity of VD-hemopressin (α) for EA.

### 3.5 Microinjection of RVD-hemopressin (α) into the vlPAG mimicked the EA effect on pain hypersensitivity

A microinjection of RVD-hemopressin (α) (1 nmol, 2 nmol, 5 nmol; 0.5 µL) into the vlPAG markedly increased the mechanical withdrawal thresholds of the KOA mice (*p* < 0.05), as well as the EA group (*p* < 0.05). As shown in Figure 4C, the time course curves (i.e., KOA + Saline, EA, and RVD-hemopressin (α) groups) were significantly different between treatments (F_(4, 343)_ = 28.59; *p* < 0.0001), across times (F_(6, 343)_ = 1249.47; *p* < 0.0001), and for their interactions (F_(24, 343)_ = 6.22; *p* < 0.0001). Further analyses showed that the mechanical thresholds in the 1 nmol and 2 nmol RVD-hemopressin (α) groups were significantly higher than those in the KOA + Saline group from the 25th to the 27th day after KOA induction (*p* < 0.05). The mechanical thresholds in the 5 nmol RVD-hemopressin (α) group were significantly higher than those in the KOA + Saline group from the 23rd to the 27th day after KOA induction, as well as those in the EA group (*p* < 0.05). In addition, the analgesic effects induced by 5 nmol RVD-hemopressin (α) on the mechanical thresholds were significantly higher than those in the 1 nmol RVD-hemopressin (α) and the 2 nmol RVD-hemopressin (α) (*p* < 0.05) groups from the 23rd to 25th day after KOA induction. The mechanical thresholds in the 1 nmol RVD-hemopressin (α) group were significantly lower than those in the EA group on the 25th day after KOA induction (*p* < 0.05). No significant difference was observed between the 5 nmol RVD-hemopressin (α) group and the EA group from the 23rd to the 27th day after KOA induction (*p* > 0.05; Figure 4C), signifying the occurrence of similar roles of RVD-hemopressin at 5 nmol concentration and EA.

Microinjection of RVD-hemopressin (α) (1 nmol, 2 nmol, 5 nmol; 0.5 µL) into the vlPAG also increased the thermal withdrawal latency (*p* < 0.05). As shown in Figure 4D, the time course curves (i.e., KOA + Saline, EA, and RVD-hemopressin (α) groups) were significantly different between treatments (F_(4, 280)_ = 39.73; *p* < 0.0001), across times (F_(6, 280)_ = 75.55; *p* < 0.0001), and for their interactions (F_(24, 280_ = 6.03; *p* < 0.0001). Further analyses showed that the withdrawal latency in the 1 nmol, 2 nmol, and 5 nmol RVD-hemopressin (α) groups was significantly higher than the KOA + Saline group from the 21st to the 27th day after KOA induction, as well as the EA group (*p* < 0.05). In addition, the analgesic effects induced by EA and 5 nmol RVD-hemopressin (α) on the withdrawal latency were significantly higher than the 1 nmol RVD-hemopressin (α) group on the 27th day after KOA induction (*p* < 0.05). No significant difference was detected between the 2 nmol and 5 nmol RVD-hemopressin (α) groups and the EA group from the 21st to the 27th day after KOA induction (*p* > 0.05; Figure 4D).

### 3.6 Microinjection of the CB1 receptor antagonist AM251 into the vlPAG reversed the VD-hemopressin (α) effect on pain hypersensitivity

**FIGURE 5:**
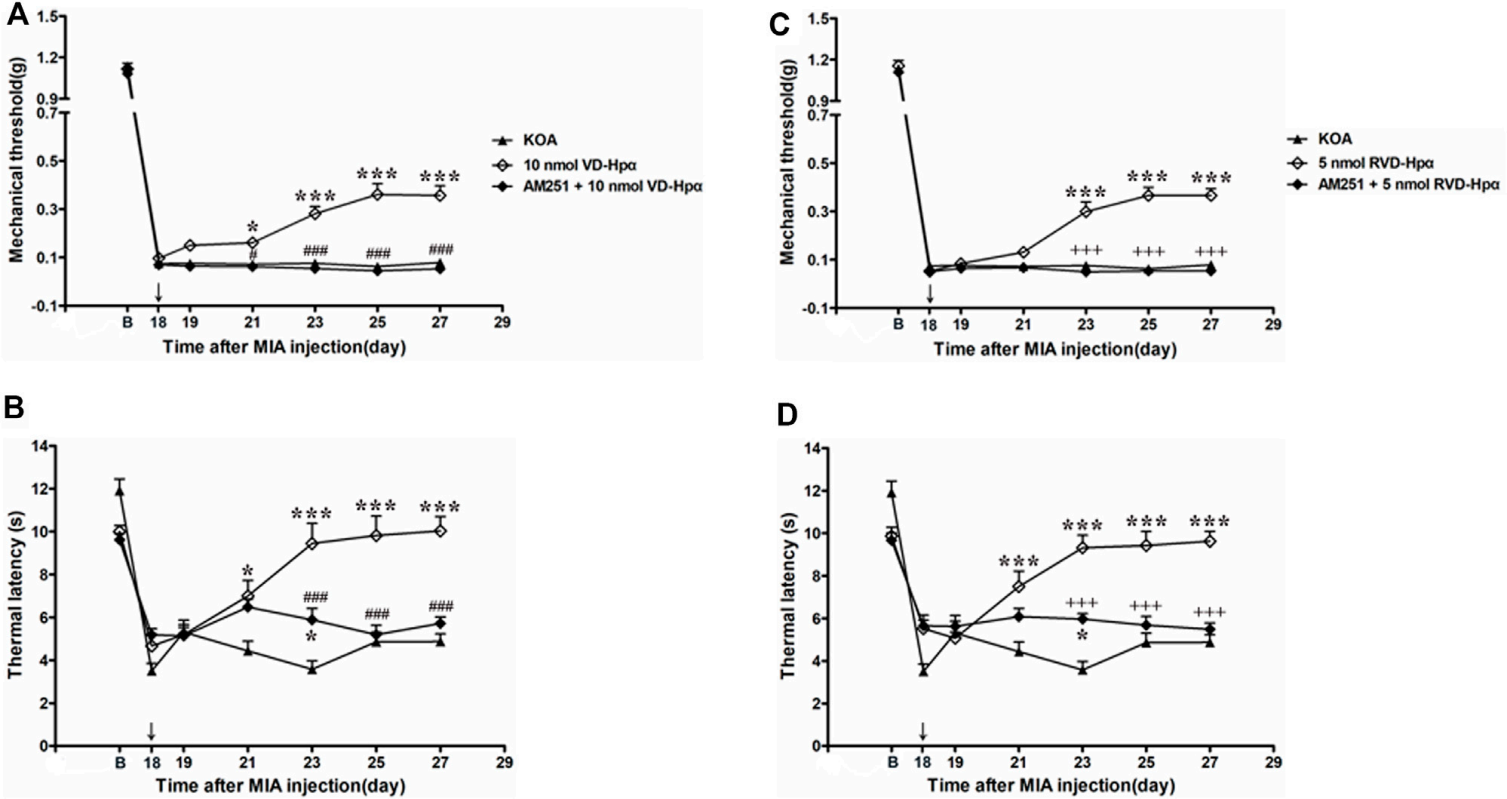
Effects of the CB1 receptor antagonist AM251 on VD-hemopressin (α) and RVD-hemopressin (α) analgesia. **(A, B)** Time course of the effect of the CB1 receptor antagonist AM251 on tactile and thermal withdrawal thresholds of VD-hemopressin (α) mice. **(C, D)** Time course of the effect of the CB1 receptor antagonist AM251 on tactile and thermal withdrawal thresholds of RVD-hemopressin (α) mice. The CB1 receptor antagonist AM251 was microinjected into the vlPAG 10 min before VD-hemopressin (α) or RVD-hemopressin (α) starting from 18 days after MIA injection, once every other day for five times, as indicated by the black arrow. *p < 0.05; ***p < 0.001, compared with KOA group; ###*p* < 0.001, compared with the 10 nmol VD-hemopressin (α) group; +++ *p* < 0.001, compared with the 5-nmol RVD-hemopressin (α) group. Data are expressed as means ± SEM (n = 5–11 mice in each group).

To identify whether the CB1 receptor was involved in the analgesic effect of VD-hemopressin (α), the CB1 receptor selective antagonist AM251 (20 nmol/0.5 µL) was administered 10 min before injection of 10 nmol VD-hemopressin (α). The AM251 was found to reverse the VD-hemopressin (α) effect on the tactile withdrawal thresholds (*p* < 0.001). As shown in Figure 5A, the time course curves (i.e., KOA, 10 nmol VD-hemopressin (α), and AM251 + 10 nmol VD-hemopressin (α) groups) were significantly different between treatments (F_(2, 189)_ = 93.34; *p* < 0.0001), across times (F_(6, 189)_ = 750.8; *p* < 0.0001), and for their interactions (F_(12, 189)_ = 9.961; *p* < 0.0001). Further analyses showed that the mechanical thresholds in the AM251 + 10 nmol VD-hemopressin (α) group were significantly less than those in the AM251 group from the 21st to the 27th day after KOA induction (*p* < 0.05; Figure 5A). No significant difference was measured between the AM251 + 10 nmol VD-hemopressin (α) group and the KOA group (*p* > 0.05; Figure 5A).

Similarly, the AM251 also reversed the 10 nmol VD-hemopressin (α) effect on the thermal withdrawal latency (*p* < 0.001). As shown in Figure 5B, the time course curves (i.e., KOA, 10 nmol VD-hemopressin (α), and AM251 + 10 nmol VD-hemopressin (α) groups) were significantly different between treatments (F_(2, 126)_ = 44.66; *p* < 0.0001), across times (F_(6, 126)_ = 43.67; *p* < 0.0001), and for their interactions (F_(12, 126)_ = 10.12; *p* < 0.0001). Further analyses showed that the withdrawal latency in the AM251 + 10 nmol VD-hemopressin (α) group was significantly less than the AM251 group from the 23rd to the 27th day after KOA induction (*p* < 0.05; Figure 5B). The withdrawal latency in the AM251 + 10 nmol VD-hemopressin (α) group was also significantly higher than in the KOA group on the 23rd day after KOA induction (*p* < 0.05; Figure 5B).

### 3.7 Microinjection of the CB1 receptor antagonist AM251 into the vlPAG reversed the RVD-hemopressin (α) effect on pain hypersensitivity

We microinjected the CB1 receptor antagonist AM251 (20 nmol) into the vlPAG to determine whether the CB1 receptor contributes to the RVD-hemopressin (α) effect on pain hypersensitivity in KOA mice. The AM251 reversed the RVD-hemopressin (α) effect on the tactile withdrawal thresholds (*p* < 0.001). As shown in Figure 5C, the time course curves (i.e., KOA, 5 nmol RVD-hemopressin (α), and AM251 + 5 nmol RVD-hemopressin (α) groups) were significantly different between treatments (F_(2, 182)_ = 96.61; *p* < 0.0001), across times (F_(6, 182)_ = 1001.47; *p* < 0.0001), and for their interactions (F_(12, 182)_ = 15.28; *p* < 0.0001). Further analyses showed that the mechanical thresholds in the AM251 + 5 nmol RVD-hemopressin (α) group were significantly less than those in the AM251 group from the 23rd to the 27th day after KOA induction (*p* < 0.001; Figure 5C). No significant difference was measured between the AM251 + 5 nmol RVD-hemopressin (α) group and the KOA group (*p* > 0.05; Figure 5C).

Similarly, the application of the AM251 into the vlPAG reversed the 5 nmol RVD-hemopressin (α) effect on the thermal withdrawal latency (*p* < 0.001). As shown in Figure 5D, the time course curves (i.e., KOA, 5 nmol RVD-hemopressin (α), and AM251 + 5 nmol RVD-hemopressin (α) groups) were significantly different between treatments (F_(2, 140)_ = 47.80; *p* < 0.0001), across times (F_(6, 140)_ = 37.55; *p* < 0.0001), and for their interactions (F_(12, 140)_ = 9.409; *p* < 0.0001). Further analyses showed that the withdrawal latency in the AM251 + 5 nmol RVD-hemopressin (α) group was significantly less than the AM251 group from the 23rd to the 27th day after KOA induction (*p* < 0.05; Figure 5D). No significant difference was measured between the AM251 + 5 nmol RVD-hemopressin (α) group and the KOA group from the 25th to the 27th day after KOA induction (*p* > 0.05; Figure 5D). The withdrawal latency in the AM251 + 5 nmol RVD-hemopressin (α) group was also significantly higher than in the KOA group on the 23rd day after KOA induction (*p* < 0.05; Figure 5D).

### 3.8 EA increased the 26S proteasome expression in the PAG of KOA mice

**FIGURE 6:**
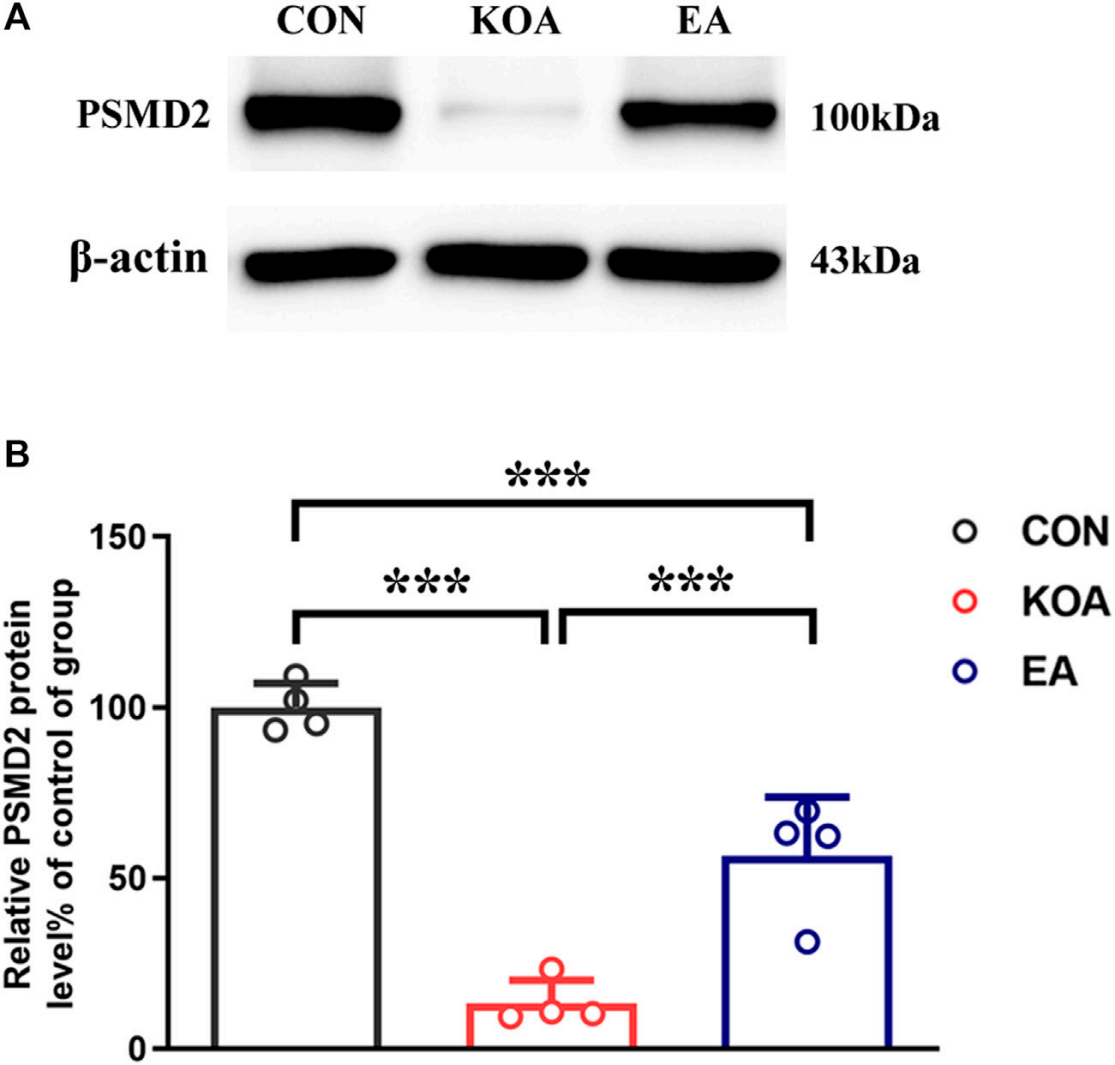
Quantitative analysis of the protein level of 26S proteasome (PSMD2) in the PAG tissues. **(A)** The representative gel image shows the protein level of the PSMD2 in PAG tissues obtained from control (CON), KOA, and KOA treated with EA. β-actin was used as a loading control. The protein band at 100 kDa corresponds to the PSMD2. **(B)** Summary data show the effect of KOA and EA on the protein level of the PSMD2 in PAG tissues. Data are expressed as means ± SEM (n = 4 mice in each group). ***p < 0.001.

Studies have reported hemoglobin degradation by proteasome activity after ubiquitination or oxidation in erythrocytes (Pacifici et al., 1993; Wenzel and Baumeister, 1993). Moreover, the 26S proteasome is known to generate peptides ranging from 3 to 22 amino acids (Kisselev et al., 1999) and is consistent with the size of VD-hemopressin (α) and RVD-hemopressin (α). Thus, we used Western blotting to evaluate the effect of EA on the 26S proteasome protein level. The 26S proteasome protein bands were presented in the PAG tissues (Figure 6A). Compared with the control group, the 26S proteasome protein level was significantly reduced in the PAG of KOA mice (*p* < 0.001; Figure 6B). EA significantly increased the 26S proteasome level compared to the KOA group (*p* < 0.001; Figure 6B).

### 3.9 vlPAG microinjection of the 26S proteasome inhibitor MG132 reversed the EA effects on pain hypersensitivity and upregulated the concentration of RVD-hemopressin (α)

**FIGURE 7:**
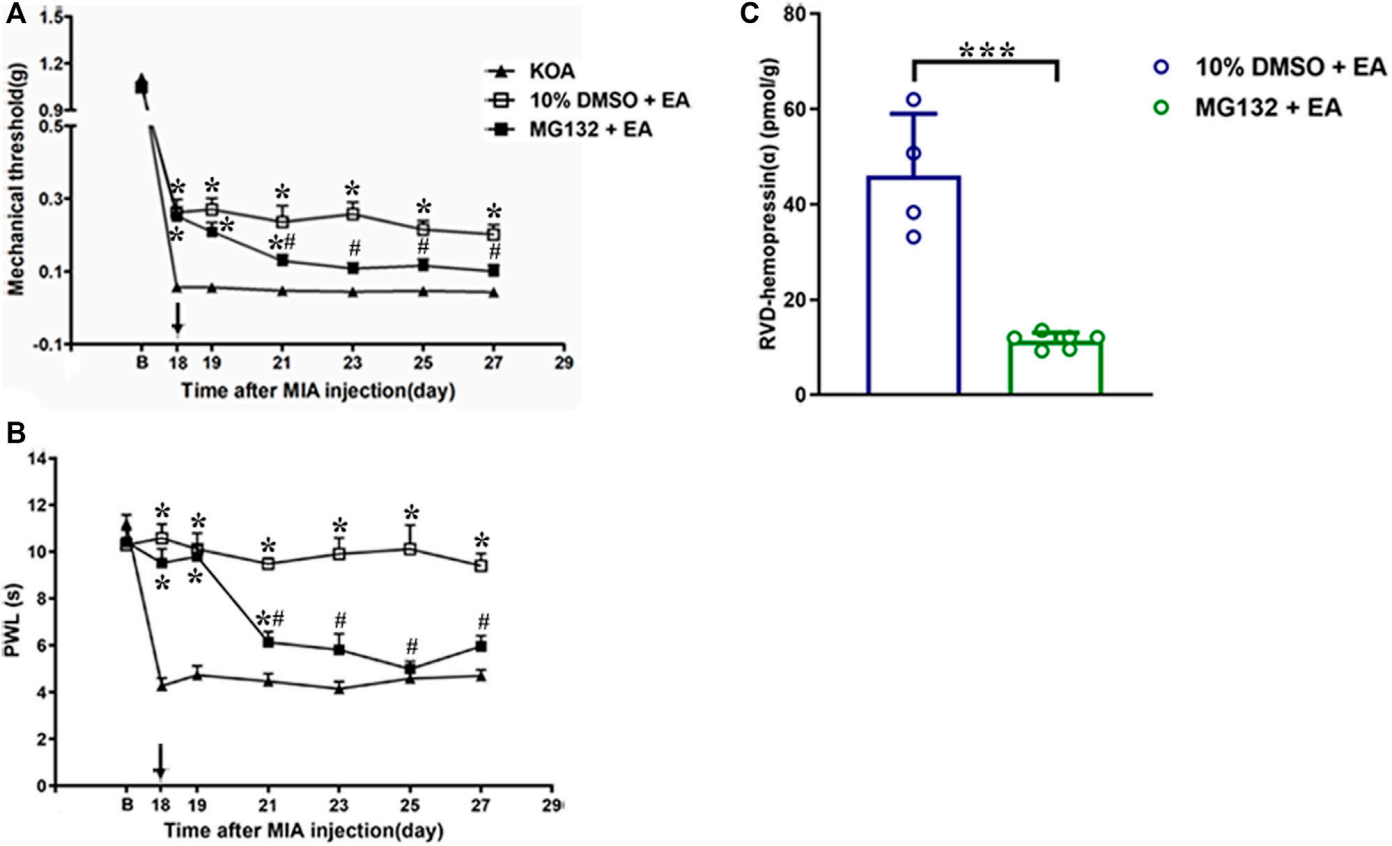
Effects of the 26S proteasome inhibitor MG132 on EA analgesia and RVD-hemopressin (α) concentration in the PAG. **(A, B)** Time course of the effect of the 26S proteasome inhibitor MG132 on tactile and thermal withdrawal thresholds of EA mice. EA was administered for 30 min, once every other day for 4 weeks, starting from 2 days after the MIA injection. The 26S proteasome inhibitor MG132 (4 μg) was microinjected into the vlPAG 30 min before EA starting from 18 days after MIA injection, once every other day for five times, as indicated by the black arrow. Data are expressed as means ± SEM (n = 8–11 mice in each group). *p < 0.05, compared with the KOA group; #*p* < 0.05, compared with the 10% DMSO + EA group. **(C)** Summary data show the concentration of RVD-hemopressin (α) in the PAG. Data are expressed as means ± SEM (n = 4–6 mice in each group). *p < 0.001.

To explore whether the 26S proteasome was involved in the degradation of hemoglobin, the 26S proteasome inhibitor MG132 (0.4 μg/0.5 µL) was administered 30 min before EA starting from the 18th day after KOA induction, once every other day for five times (Yang et al., 2008). The MG132 reversed the EA effect on the tactile withdrawal thresholds (*p* < 0.001). As shown in Figure 7A, the time course curves (i.e., KOA, MG132 + EA, and 10% DMSO + EA groups) were significantly different between treatments (F_(2, 189)_ = 93.17; *p* < 0.0001) across times (F_(6, 189)_ = 843.88; *p* < 0.0001), and for their interactions (F_(12, 189)_ = 7.39; *p* < 0.0001). Further analyses showed that the mechanical thresholds in the MG132 + EA group were significantly less than those in the 10% DMSO + EA group from the 21st to the 27th day after KOA induction (*p* < 0.05; Figure 7A). No significant difference was measured between the MG132 + EA and KOA groups from the 23rd to the 27th day after KOA induction (*p* > 0.05; Figure 7A).

Similarly, the MG132 reversed the EA effect on the thermal withdrawal latency (*p* < 0.001). As shown in Figure 7B, the time course curves (i.e., KOA, MG132 + EA, and 10% DMSO + EA groups) were significantly different between treatments (F_(2, 189)_ = 145.56; *p* < 0.0001) across times (F_(6, 189)_ = 28.87; *p* < 0.0001), and for their interactions (F_(12, 189)_ = 11.04; *p* < 0.0001). Further analyses showed that the withdrawal latency in the MG132 + EA group was significantly less than in the 10% DMSO + EA group from the 21st to the 27th day after KOA induction (*p* < 0.05; Figure 7B). No significant difference was measured between the MG132 + EA group and the KOA group from the 23rd to the 27th day after KOA induction (*p* > 0.05; Figure 7B). These results signify that MG132 reversed the ameliorating effect of EA on pain hypersensitivity.

We further examined the concentration of VD-hemopressin (α) and RVD-hemopressin (α). The result showed that microinjection of MG132 before EA prevented the effect of EA upregulation of the concentration of RVD-hemopressin (α) in the PAG (*p* < 0.001; Figure 7C), but VD-hemopressin (α) could not be detected in the MG132 + EA group.

### 3.10 vlPAG microinjection of the 26S proteasome inhibitor MG132 increased the expression of hemoglobin α-chain

**FIGURE 8:**
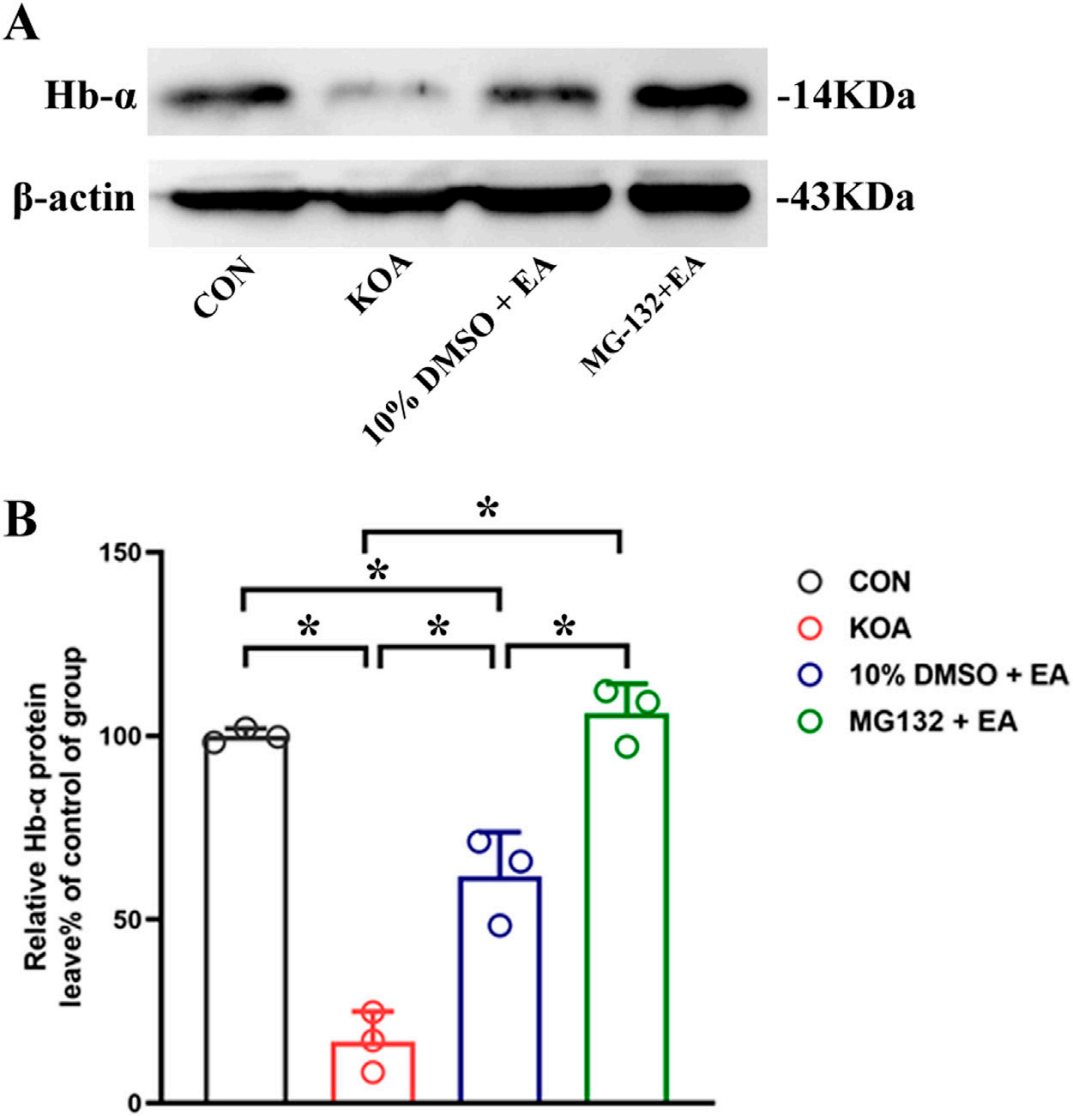
Effects of the 26S proteasome inhibitor MG132 on hemoglobin α-chain expression in the PAG. **(A)** The representative gel image shows the protein level of hemoglobin α-chain in PAG tissues obtained from CON, KOA, 10% DMSO + EA, and MG132 + EA groups. β-actin was used as a loading control. The protein band at 14 kDa corresponds to the hemoglobin α-chain. **(B)** Summary data show the effect of 26S proteasome inhibitor MG132 on the protein level of the hemoglobin α-chain in PAG tissues. EA was administered for 30 min, once every other day for 4 weeks, starting from 2 days after MIA injection. The 26S proteasome inhibitor MG132 (4 μg) was microinjected into the vlPAG 30 min before EA starting from 18 days after MIA injection, once every other day for five times. Data are expressed as means ± SEM (n = 3 mice in each group). *p < 0.05.

Because VD-hemopressin (α) and RVD-hemopressin (α) peptides are derived from the hemoglobin α chain, we also investigated detecting the expression of the hemoglobin α chain in the PAG. The result showed that compared with the 10% DMSO + EA group, the PSMD2 protein level was significantly increased in the MG132 + EA group (*p* < 0.05; Figures 8A, B). These results indicated that EA might produce VD-hemopressin (α) and RVD-hemopressin (α) by promoting 26S proteasome to degrade the hemoglobin α chain, thereby exerting an analgesic effect.

## 4 Discussion

The present study utilized RNA-seq to evaluate the involvement of differentially expressed genes *Hba-a1* and *Hba-a2*, which encode the hemoglobin α chain, in EA-induced analgesia. It was demonstrated that EA treatment of 2 Hz + 1 mA significantly increased the levels of hemoglobin α chain and its active-derived peptides VD-hemopressin (α) and RVD-hemopressin(α) in KOA mice. Microinjection of VD-hemopressin (α) and RVD-hemopressin (α) into the vlPAG mimicked the analgesic effect of EA, while microinjection of AM251 reversed this effect. Additionally, EA significantly increased the expression of 26S proteasome in the PAG, and microinjection of 26S proteasome inhibitor MG132 reversed the EA effects on pain hypersensitivity and upregulated the concentration of RVD-hemopressin (α). Meanwhile, we found that microinjection of MG132 before EA upregulated the expression of the hemoglobin α chain, indicating that it inhibited the degradation of the hemoglobin α chain. Our study provided evidence that EA upregulated the concentration of VD-hemopressin (α) and RVD-hemopressin (α) through the promotion of hemoglobin α chain degradation by 26S proteasome and activation of CB1 receptor, thereby exerting chronic pain inhibition in a mouse model of KOA.

EA has been widely used for alleviating diverse pains under morbid conditions (Chen et al., 2022; Li et al., 2019; Liao and Lin, 2021). However, the central mechanism by which EA modifies nociception is still a conundrum. In the present study, RNA-seq was used to obtain transcriptome data from control, KOA, and EA groups to explore EA-induced analgesia-related DEGs and their central regulatory mechanisms. Clustering analysis of DEGs showed that 10 genes were downregulated in the KOA group while upregulated in the EA group, and 12 genes were upregulated in the KOA group while downregulated in the EA group. Among these DEG data, two genes, Hba-a1 and Hba-a2, were selected to be explored because the gene *Hba-a2* has the largest fold change, and *Hba-a1* has more than three times change compared with the control group. The *Hba-a1* and *Hba-a2* encode for hemoglobin α chain, which, upon degradation, yields cannabinopeptide ligands. We have been considering the role of the endocannabinoid system in EA analgesia. Therefore, we used RT-qPCR to explore it further for verification in this study. Interestingly, it was found that the expressions of *Hba-a1* and *Hba-a2* in the PAGs of KOA mice were significantly downregulated compared with the control group, and EA significantly increased the expression of *Hba-a1* and *Hba-a2*, which was consistent with the results of RNA-seq. Note that *Hba-a1* expression was notably more downregulated than *Hba-a2* in the KOA group. This may be caused by the different expression levels of *Hba-a1* and *Hba-a2* in the mice. These data indicate the involvement of *Hba-a1* and *Hba-a2* genes in EA-mediated analgesia.

It is established that *Hba-a1* and *Hba-a2* encode the hemoglobin α chain. As commonly understood, the hemoglobin α chain is an important component of hemoglobin, and it has long been thought that hemoglobin expression is restricted to erythrocytes and precursor cells of the erythroid lineage (Russo et al., 2013). However, recent studies have discovered that hemoglobin α also expressed in neurons of the midbrain and cortex, cerebellum, hippocampus, and striatum (Biagioli et al., 2009; Richter et al., 2009; Russo et al., 2013; Schelshorn et al., 2009). Similarly, the present study has found a significant increase in the protein level of the hemoglobin α chain in the PAG following repeated EA treatments. We further analyzed the correlation between the expression of *Hba-a1* and *Hba-a2* and hemoglobin α chain protein expression and observed a good correlation between them (r_*Hba-a1*_ = 0.9169, *P*_*Hba-a1*_ = 0.0005; r_*Hba-a2*_ = 0.8451, *P*_*Hba-a2*_ = 0.0041, Supplementary Figure S1). Thus, these data indicate that the hemoglobin α chain is involved in EA analgesia, but its mechanism remains unclear.

It has been demonstrated that VD-hemopressin (α) and RVD-hemopressin (α) peptides are derived from the hemoglobin α chain in mouse brains (Gomes et al., 2009). In this study, we have shown that the levels of VD-hemopressin (α) and RVD-hemopressin (α) were significantly increased after EA treatments. Further studies have shown that the CB1 receptor mediated the analgesic effect of VD-hemopressin (α), but whether RVD-hemopressin (α) has an analgesic effect is unclear (Gomes et al., 2009; Han et al., 2014; Zheng et al., 2017). To investigate this question, 10 nmol of VD-hemopressin (α) and various doses of RVD-hemopressin (α) were microinjected into the vlPAG, and a similar effect was observed mimicking EA treatment-induced analgesia. Moreover, the CB1 receptor selective antagonist AM251 reversed the analgesic effect of VD-hemopressin (α) and RVD-hemopressin (α). These results indicate that EA upregulated the concentration of VD-hemopressin (α) and RVD-hemopressin (α) in the PAG and activated the CB1 receptor to inhibit chronic pain in a mouse model of KOA.

It will be interesting to explore the mechanisms involved in generating VD-hemopressin (α) and RVD-hemopressin (α). Previous studies have demonstrated that the 26S proteasome is known to generate peptides ranging from 3 to 22 amino acids long (Kisselev et al., 1999; Yuan T. et al., 2018). Furthermore, the VD-hemopressin (α) and RVD-hemopressin (α) are within the appropriate size range for 26S proteasome products. Therefore, one possibility is that VD-hemopressin (α) and RVD-hemopressin (α) may be produced through 26S proteasome degradation of the hemoglobin α chain in neurons.

The present study further revealed that EA significantly increased the expression of 26S proteasome in the PAG. Moreover, microinjection of 26S proteasome inhibitor MG132 into the vlPAG before EA reversed both the anti-allodynic effect and upregulation of the concentration of RVD-hemopressin (α) by EA treatment, but VD-hemopressin (α) was not detected. It was earlier documented that RVD-hemopressin (α) is the most frequently detected α-hemoglobin-derived peptide by many folds, making it the most abundant neuropeptide (Gomes et al., 2009). Cleavage of hemopressin at the D-P bond would give rise to the shorter peptides. Therefore, it is reasonable to suggest that VD-hemopressin (α) could not be detected after microinjection of 26S proteasome inhibitor MG132 before EA because VD-hemopressin (α) degradation most likely produced other shorter peptides. Microinjection of the 26S proteasome inhibitor MG132 into the vlPAG before EA also significantly increased the expression of the hemoglobin α chain. These results provide support to the hypothesis that VD-hemopressin (α) and RVD-hemopressin (α) are produced through 26S proteasome degradation of the hemoglobin α chain.

## 5 Conclusion

Taken together, the present study demonstrated that the hemoglobin α chain in the PAG is involved in EA analgesia. A possible mechanism underlying this effect is that EA enhances the concentration of VD-hemopressin (α) and RVD-hemopressin (α) via the promotion of the hemoglobin α chain degradation by the 26S proteasome and activates the CB1 receptor, which brings about chronic pain inhibition in a mouse model of KOA.

## Data Availability

The data presented in this study are deposited in the Sequence Read Archive (SRA) repository (https://www.ncbi.nlm.nih.gov/sra/), accession numbers: SRR30787944, SRR30787945, SRR30787946, SRR30787947, SRR30787948, SRR30787949, SRR30787950, SRR30787951, SRR30787952.

## Abbreviations

2-AG, 2-araehidonoyl glycerol; AEA, arachidonic ethanolamine anandamide; EA, electroacupuncture; KOA, knee osteoarthritis; MIA, monosodium iodoacetate; PAG, periaqueductal gray; vlPAG, ventrolateral pariaqueducta gray.

## References

[B1] Bauer M. Chicca A. Tamborrini M. Eisen D. Lerner R. Lutz B. (2012). Identification and quantification of a new family of peptide endocannabinoids (Pepcans) showing negative allosteric modulation at CB1 receptors. J. Biol. Chem. 287 (44), 36944–36967. 10.1074/jbc.M112.382481 22952224 PMC3481297

[B2] Biagioli M. Pinto M. Cesselli D. Zaninello M. Lazarevic D. Roncaglia P. (2009). Unexpected expression of alpha- and beta-globin in mesencephalic dopaminergic neurons and glial cells. Proc. Natl. Acad. Sci. U. S. A. 106 (36), 15454–15459. 10.1073/pnas.0813216106 19717439 PMC2732704

[B3] Chaplan S. R. Bach F. W. Pogrel J. W. Chung J. M. Yaksh T. L. (1994). Quantitative assessment of tactile allodynia in the rat paw. J. Neurosci. Methods 53 (1), 55–63. 10.1016/0165-0270(94)90144-9 7990513

[B4] Chen L. Zhang J. Li F. Qiu Y. Wang L. Li Y. H. (2009). Endogenous anandamide and cannabinoid receptor-2 contribute to electroacupuncture analgesia in rats. J. Pain 10 (7), 732–739. 10.1016/j.jpain.2008.12.012 19409856

[B5] Chen Y. Zhou Y. Li X. C. Ma X. Mi W. L. Chu Y. X. (2022). Neuronal GRK2 regulates microglial activation and contributes to electroacupuncture analgesia on inflammatory pain in mice. Biol. Res. 55 (1), 5. 10.1186/s40659-022-00374-6 35115050 PMC8812183

[B6] de Hoon M. J. Imoto S. Nolan J. Miyano S. (2004). Open source clustering software. Bioinformatics 20 (9), 1453–1454. 10.1093/bioinformatics/bth078 14871861

[B7] Eisen M. B. Spellman P. T. Brown P. O. Botstein D. (1998). Cluster analysis and display of genome-wide expression patterns. Proc. Natl. Acad. Sci. U. S. A. 95 (25), 14863–14868. 10.1073/pnas.95.25.14863 9843981 PMC24541

[B8] Fernihough J. Gentry C. Malcangio M. Fox A. Rediske J. Pellas T. (2004). Pain related behaviour in two models of osteoarthritis in the rat knee. Pain 112 (1-2), 83–93. 10.1016/j.pain.2004.08.004 15494188

[B9] Gelman J. S. Fricker L. D. (2010). Hemopressin and other bioactive peptides from cytosolic proteins: are these non-classical neuropeptides? AAPS J. 12 (3), 279–289. 10.1208/s12248-010-9186-0 20383670 PMC2895439

[B10] Gomes I. Grushko J. S. Golebiewska U. Hoogendoorn S. Gupta A. Heimann A. S. (2009). Novel endogenous peptide agonists of cannabinoid receptors. FASEB J. 23 (9), 3020–3029. 10.1096/fj.09-132142 19380512 PMC2735371

[B11] Han Z. L. Fang Q. Wang Z. L. Li X. H. Li N. Chang X. M. (2014). Antinociceptive effects of central administration of the endogenous cannabinoid receptor type 1 agonist VDPVNFKLLSH-OH [(m)VD-hemopressin(α)], an N-terminally extended hemopressin peptide. J. Pharmacol. Exp. Ther. 348 (2), 316–323. 10.1124/jpet.113.209866 24307201

[B12] Harvey V. L. Dickenson A. H. (2009). Behavioural and electrophysiological characterisation of experimentally induced osteoarthritis and neuropathy in C57Bl/6 mice. Mol. Pain 5, 18. 10.1186/1744-8069-5-18 19379487 PMC2678995

[B13] Jiang Z. Li Y. Wang Q. Fang Z. Deng J. Zhang X. (2022). Combined-acupoint electroacupuncture induces better analgesia via activating the endocannabinoid system in the spinal cord. Neural Plast. 2022, 7670629. 10.1155/2022/7670629 36160326 PMC9499800

[B14] Kisselev A. F. Akopian T. N. Woo K. M. Goldberg A. L. (1999). The sizes of peptides generated from protein by mammalian 26 and 20 S proteasomes. Implications for understanding the degradative mechanism and antigen presentation. J. Biol. Chem. 274 (6), 3363–3371. 10.1074/jbc.274.6.3363 9920878

[B15] La Porta C. Bura S. A. Aracil-Fernandez A. Manzanares J. Maldonado R. (2013). Role of CB1 and CB2 cannabinoid receptors in the development of joint pain induced by monosodium iodoacetate. Pain 154 (1), 160–174. 10.1016/j.pain.2012.10.009 23199705

[B16] Leone S. Ferrante C. Recinella L. Chiavaroli A. Mollica A. Tomboly C. (2018). Effects of RVD-hemopressin (α) on feeding and body weight after standard or cafeteria diet in rats. Neuropeptides 72, 38–46. 10.1016/j.npep.2018.10.002 30396596

[B17] Li Y. Yin C. Li X. Liu B. Wang J. Zheng X. (2019). Electroacupuncture alleviates paclitaxel-induced peripheral neuropathic pain in rats via suppressing TLR4 signaling and TRPV1 upregulation in sensory neurons. Int. J. Mol. Sci. 20 (23), 5917. 10.3390/ijms20235917 31775332 PMC6929119

[B18] Liao H. Y. Lin Y. W. (2021). Electroacupuncture attenuates chronic inflammatory pain and depression comorbidity through transient receptor potential V1 in the brain. Am. J. Chin. Med. 49 (6), 1417–1435. 10.1142/S0192415X2150066X 34224338

[B19] Luan L. Bousie J. Pranata A. Adams R. Han J. (2021). Stationary cycling exercise for knee osteoarthritis: a systematic review and meta-analysis. Clin. Rehabil. 35 (4), 522–533. 10.1177/0269215520971795 33167714

[B20] Lv Z. T. Shen L. L. Zhu B. Zhang Z. Q. Ma C. Y. Huang G. F. (2019). Effects of intensity of electroacupuncture on chronic pain in patients with knee osteoarthritis: a randomized controlled trial. Arthritis Res. Ther. 21 (1), 120. 10.1186/s13075-019-1899-6 31088511 PMC6518678

[B21] Ng M. M. Leung M. C. Poon D. M. (2003). The effects of electro-acupuncture and transcutaneous electrical nerve stimulation on patients with painful osteoarthritic knees: a randomized controlled trial with follow-up evaluation. J. Altern. Complement. Med. 9 (5), 641–649. 10.1089/107555303322524490 14629842

[B22] Pacifici R. E. Kono Y. Davies K. J. (1993). Hydrophobicity as the signal for selective degradation of hydroxyl radical-modified hemoglobin by the multicatalytic proteinase complex, proteasome. J. Biol. Chem. 268 (21), 15405–15411. 10.1016/s0021-9258(18)82272-4 8393440

[B23] Richter F. Meurers B. H. Zhu C. Medvedeva V. P. Chesselet M. F. (2009). Neurons express hemoglobin alpha- and beta-chains in rat and human brains. J. Comp. Neurol. 515 (5), 538–547. 10.1002/cne.22062 19479992 PMC3123135

[B24] Russo R. Zucchelli S. Codrich M. Marcuzzi F. Verde C. Gustincich S. (2013). Hemoglobin is present as a canonical α2β2 tetramer in dopaminergic neurons. Biochim. Biophys. Acta 1834 (9), 1939–1943. 10.1016/j.bbapap.2013.05.005 23685348

[B25] Saldanha A. J. (2004). Java Treeview--extensible visualization of microarray data. Bioinformatics 20 (17), 3246–3248. 10.1093/bioinformatics/bth349 15180930

[B26] Schelshorn D. W. Schneider A. Kuschinsky W. Weber D. Kruger C. Dittgen T. (2009). Expression of hemoglobin in rodent neurons. J. Cereb. Blood Flow. Metab. 29 (3), 585–595. 10.1038/jcbfm.2008.152 19116637

[B27] Selfe T. K. Taylor A. G. (2008). Acupuncture and osteoarthritis of the knee: a review of randomized, controlled trials. Fam. Community Health 31 (3), 247–254. 10.1097/01.FCH.0000324482.78577.0f 18552606 PMC2810544

[B28] Tai H. C. Schuman E. M. (2008). Ubiquitin, the proteasome and protein degradation in neuronal function and dysfunction. Nat. Rev. Neurosci. 9 (11), 826–838. 10.1038/nrn2499 18931696

[B29] Wenzel T. Baumeister W. (1993). Thermoplasma acidophilum proteasomes degrade partially unfolded and ubiquitin-associated proteins. FEBS Lett. 326 (1-3), 215–218. 10.1016/0014-5793(93)81793-y 8391997

[B30] Yang L. Wang S. Lim G. Sung B. Zeng Q. Mao J. (2008). Inhibition of the ubiquitin-proteasome activity prevents glutamate transporter degradation and morphine tolerance. Pain 140 (3), 472–478. 10.1016/j.pain.2008.09.028 18986766 PMC3454498

[B31] Yuan T. Yan F. Ying M. Cao J. He Q. Zhu H. (2018a). Inhibition of ubiquitin-specific proteases as a novel anticancer therapeutic strategy. Front. Pharmacol. 9, 1080. 10.3389/fphar.2018.01080 30319415 PMC6171565

[B32] Yuan X. C. Wang Q. Su W. Li H. P. Wu C. H. Gao F. (2018b). Electroacupuncture potentiates peripheral CB2 receptor-inhibited chronic pain in a mouse model of knee osteoarthritis. J. Pain Res. 11, 2797–2808. 10.2147/JPR.S171664 30510442 PMC6231462

[B33] Yuan X. C. Wang Y. Y. Tian L. X. Yan X. J. Guo Y. X. Zhao Y. L. (2022). Spinal 5-HT(2A) receptor is involved in electroacupuncture inhibition of chronic pain. Mol. Pain 18, 17448069221087583. 10.1177/17448069221087583 35240891 PMC9006364

[B34] Yuan X. C. Yan X. J. Tian L. X. Guo Y. X. Zhao Y. L. Baba S. S. (2021). 5-HT(7) receptor is involved in electroacupuncture inhibition of chronic pain in the spinal cord. Front. Neurosci. 15, 733779. 10.3389/fnins.2021.733779 34602973 PMC8484641

[B35] Yuan X. C. Zhu B. Jing X. H. Xiong L. Z. Wu C. H. Gao F. (2018c). Electroacupuncture potentiates cannabinoid receptor-mediated descending inhibitory control in a mouse model of knee osteoarthritis. Front. Mol. Neurosci. 11, 112. 10.3389/fnmol.2018.00112 29681797 PMC5897736

[B36] Zhang R. S. He Z. Jin W. D. Wang R. (2016). Effects of the cannabinoid 1 receptor peptide ligands hemopressin, (m)RVD-hemopressin(α) and (m)VD-hemopressin(α) on memory in novel object and object location recognition tasks in normal young and Aβ1-42-treated mice (m)RVD-hemopressin(alpha) and (m)VD-hemopressin(alpha) on memory in novel object and object location recognition tasks in normal young and Abeta1-42-treated mice. Neurobiol. Learn Mem. 134 Pt B, 264–274. 10.1016/j.nlm.2016.07.030 27481221

[B37] Zheng T. Zhang R. Zhang T. Zhang M. N. Xu B. Song J. J. (2018). CB1 cannabinoid receptor agonist mouse VD-hemopressin(α) produced supraspinal analgesic activity in the preclinical models of pain. Brain Res. 1680, 155–164. 10.1016/j.brainres.2017.12.013 29274880

[B38] Zheng T. Zhang T. Zhang R. Wang Z. L. Han Z. L. Li N. (2017). Pharmacological characterization of rat VD-hemopressin(α), an α-hemoglobin-derived peptide exhibiting cannabinoid agonist-like effects in mice. Neuropeptides 63, 83–90. 10.1016/j.npep.2016.12.006 28010996

[B39] Zimmermann M. (1983). Ethical guidelines for investigations of experimental pain in conscious animals. Pain 16 (2), 109–110. 10.1016/0304-3959(83)90201-4 6877845

