# Supplementary figures and images for: Hemoglobin α-derived peptides VD-hemopressin (α) and RVD-hemopressin (α) are involved in electroacupuncture inhibition of chronic pain
Source: Front Pharmacol. 2024 Oct 1;15:1439448. doi: 10.3389/fphar.2024.1439448 (PMC11473328; doi:10.3389/fphar.2024.1439448)

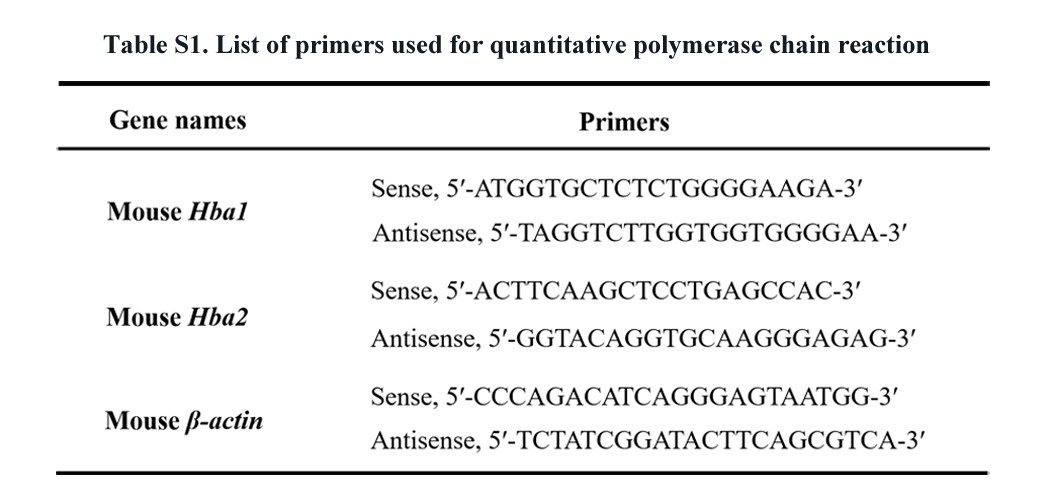

Supplement: Supplementary file 1 [file Table1.docx]

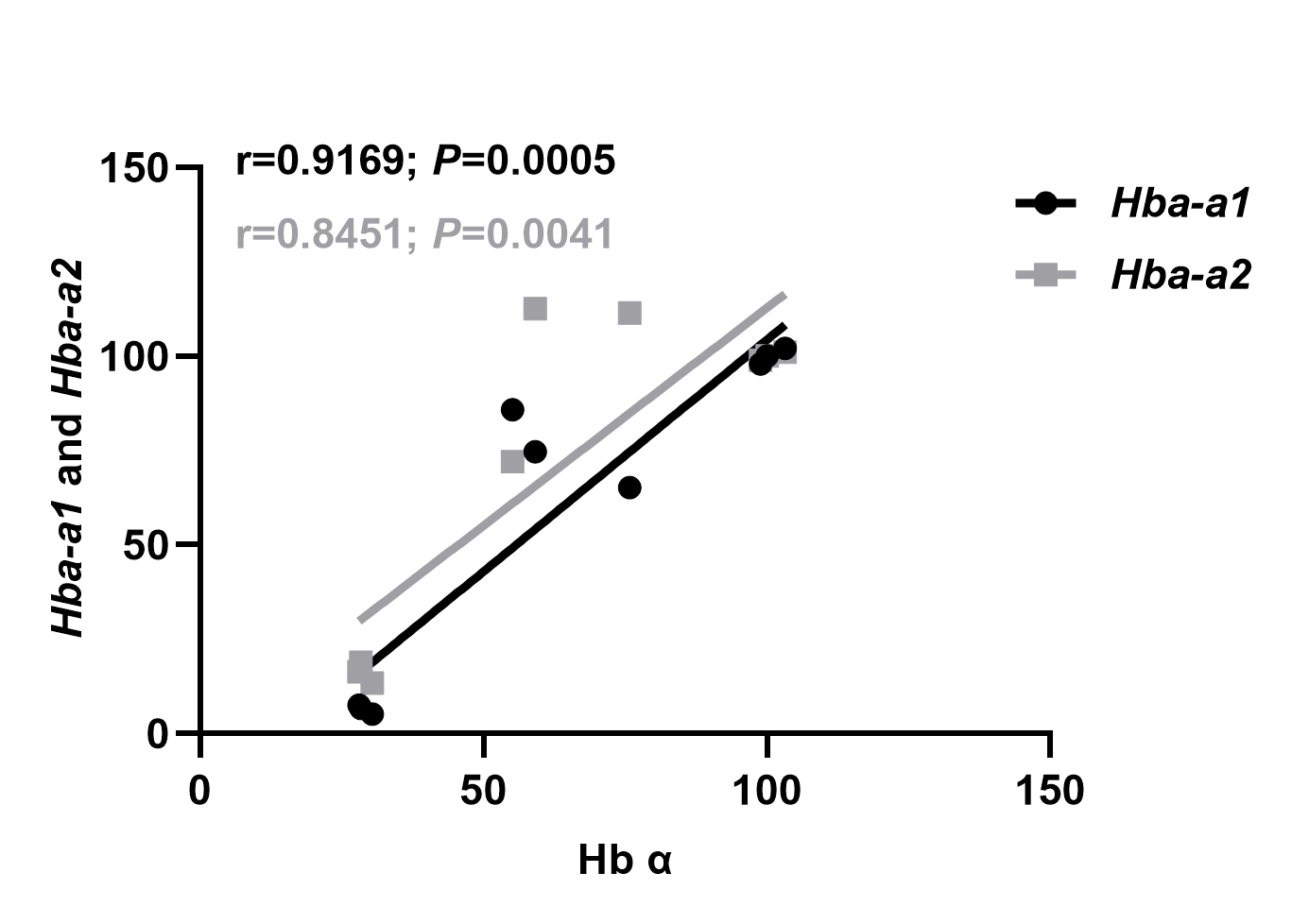

Supplement: Supplementary file 2 [file Image1.tif]
